# The Importance of Early Mycological Clearance of Uncomplicated Candidaemia and Its Implications for Clinical Practice

**DOI:** 10.1111/myc.70135

**Published:** 2025-12-05

**Authors:** O. A. Cornely, M. Bassetti, C. Garcia‐Vidal, M. Hoenigl, J. Maertens, I. Martin‐Loeches, J. P. Mira, P. L. White

**Affiliations:** ^1^ German Centre for Infection Research (DZIF), Partner Site Bonn‐Cologne Cologne Germany; ^2^ Faculty of Medicine and University Hospital Cologne Institute of Translational Research Cologne Germany; ^3^ Excellence Cluster on Cellular Stress Responses in Aging‐Associated Diseases University of Cologne Cologne Germany; ^4^ Faculty of Medicine and University Hospital Cologne, Department I of Internal Medicine Center for Integrated Oncology Aachen Bonn‐Cologne Duesseldorf Cologne Germany; ^5^ Excellence Center for Medical Mycology University of Cologne Cologne Germany; ^6^ Faculty of Medicine and University Hospital Cologne, Clinical Trials Centre Cologne (ZKS Köln) University of Cologne Cologne Germany; ^7^ Infectious Diseases Unit Policlinico San Martino Hospital, IRCCS Genoa Italy; ^8^ Department of Health Sciences (DISSAL) University of Genoa Genoa Italy; ^9^ Department of Infectious Diseases, Hospital Clinic of Barcelona‐IDIBAPS University of Barcelona Barcelona Spain; ^10^ Facultat de Medicina i Ciències de la Salut Universitat de Barcelona Spain; ^11^ Division of Infectious Diseases, Translational Medical Mycology Research Unit, European Confederation of Medical Mycology Excellence Center for Medical Mycology Medical University of Graz Graz Austria; ^12^ BioTechMed Graz Austria; ^13^ Department of Microbiology, Immunology and Transplantation KU Leuven Leuven Belgium; ^14^ Department of Haematology University Hospitals Leuven Leuven Belgium; ^15^ Department of Intensive Care Medicine, Multidisciplinary Intensive Care Research Organisation (MICRO), St James's Hospital Dublin Ireland; ^16^ School of Medicine Trinity College Dublin Dublin Ireland; ^17^ Service de Médecine Intensive and Réanimation, Hôpital Cochin, Assistance Publique‐Hôpitaux de Paris, Institut Cochin Université Paris Cité Paris France; ^18^ Institut Cochin, INSERM U1016, CNRS UMR8104 Université Paris Cité Paris France; ^19^ Public Health Wales Mycology Reference Laboratory University Hospital of Wales Cardiff UK; ^20^ Centre for Trials Research/Division of Infection and Immunity Cardiff UK

**Keywords:** antifungal agents, candidaemia, PCR, systemic infection

## Abstract

Invasive candidiasis is a life‐threatening infection associated with high morbidity, mortality and healthcare costs in both general and critical care settings. Timely diagnosis and adequate antifungal treatment are essential to improving patient outcomes and limiting unnecessary use of healthcare resources. This review explores the relationship between early *Candida* clearance with antifungal therapy, clinical outcomes, resistance patterns and economic impact. It also evaluates the role of diagnostic markers in facilitating early and accurate identification of candidaemia, enabling more precise and effective clinical management. Special attention is given to the challenges of managing candidaemia in critically ill and neutropenic patients, highlighting the need for tailored and timely interventions in these vulnerable populations.

## 1 Introduction

Invasive candidiasis (IC) is a significant cause of morbidity and mortality among immunocompromised patients, such as those with haematological malignancies, organ transplants and those in intensive care units (ICUs) [1, 2]. IC comprises both bloodstream infections (candidaemia) and deep‐seated candidiasis (DSC; with or without candidaemia), resulting from the spread of *Candida* species to a normally sterile/deep site in the body, such as the abdomen, peritoneum, bones or prosthetic implants [3, 4]. Despite advancements in antifungal treatments, candidaemia still contributes to overall mortality rates of up to 47% in certain populations [1, 2, 5, 6, 7, 8]. However, candidaemia‐attributed mortality has decreased due to improved outcomes of candidaemia caused by *Candida albicans* and *Candida parapsilosis* , while infections caused by other *Candida* species continue to show much higher attributable mortality [8]. Candidaemia imposes a significant economic burden on healthcare settings, primarily due to prolonged hospital stays [3].

Candidaemia predominantly affects critically ill and immunocompromised patients, with 60% of cases arising in ICUs [9]. Risk factors in the ICU include the presence of indwelling medical devices, extended stays and high severity scores like APACHE II [3]. Similarly, patients with haematological malignancies experience prolonged immune suppression, increasing their susceptibility to candidaemia [3]. The differences in pathophysiology and immune status between these populations underscore the need for distinct diagnostic and treatment approaches [10]. Identifying high‐risk patients early is crucial for ensuring timely antifungal therapy to reduce mortality risk.

Prompt diagnosis and treatment are crucial in managing systemic fungal infections, as delays in initiating appropriate antifungal therapy can worsen outcomes, increase mortality and prolong hospital stays [7, 11, 12, 13]. Disseminated IC is particularly challenging to manage [3]. Therefore, effective source control is essential to reduce mortality risk in IC [4, 11, 12, 13, 14, 15]. A 2005 retrospective cohort analysis demonstrated that a 12‐h delay in antifungal administration after the first positive blood culture was an independent predictor of mortality (adjusted odds ratio [OR], 2.09; 95% confidence interval [CI], 1.53–2.84; *p* = 0.018), underscoring the importance of prompt treatment and rapid diagnostics [12, 16].

The three main classes of antifungals for the treatment of candidaemia are echinocandins, azoles and polyenes [3]. Echinocandins, such as anidulafungin, caspofungin, micafungin and rezafungin, are recommended as the first‐line treatment option due to their favourable efficacy and safety profile, limited drug interactions and early fungicidal activity. Azole‐based antifungals like fluconazole are generally fungistatic in action, with the potential for higher toxicity rates and antifungal resistance [2, 3, 15, 17, 18, 19, 20, 21]. However, azoles are preferred in patients with central nervous system or intraocular *Candida* infections due to their greater penetration in the cerebrospinal fluid and vitreous humour [2, 3]. In candidaemia, treatment should continue for at least 14 days after the last negative blood culture. Physicians may switch patients from an intravenous (IV) echinocandin to oral azole once stable and if the organism is susceptible to azoles [2, 18, 22].

In terms of early mycological eradication, pooled analyses from STRIVE and ReSTORE, of patients in the ICU at the time of randomisation (*N* = 294), found that rezafungin was superior to caspofungin, with faster mycological clearance and reduced median time to negative blood cultures (18 h versus 38 h, *p* = 0.001) [23]. The earlier eradication observed with rezafungin compared with caspofungin may be attributed to its high front‐loaded dosing, achieving high plasma concentrations early in therapy. This might allow for more rapid clearance of various *Candida* species, such as *C. albicans* and *Candida glabrata*, from blood or tissue and potentially prevent the development of resistance [7, 24, 25, 26]. In vitro testing has demonstrated that rezafungin is highly effective against less common *Candida* spp. with low MIC50/90 values [27]. Rezafungin MICs were comparable with those of other echinocandins and could be regarded as a preferable therapeutic option to older echinocandins due to its reduced dosing frequency [27].

Understanding of how the timing and adequacy of antifungal therapy may influence clinical outcomes, therapeutic decisions and treatment duration in IC remains limited. Key considerations involve safely de‐escalating or discontinuing therapy without compromising its effectiveness. This review explores diagnostic methods for assessing *Candida* clearance and how adequate treatment informs personalised antifungal strategies. We also address the challenges posed by emerging resistance patterns, extended hospital stays and the necessity for antifungal stewardship.

## 2 Defining Early Mycological Clearance in Uncomplicated Candidaemia

Candidiasis incorporates both superficial and invasive/deep‐seated *Candida* infections. IC includes both bloodstream infections (candidaemia) and DSC [4] and the latter may require longer periods of antifungal therapy and/or surgical debridement [18]. Managing DSC is far more challenging than candidaemia and its mycological clearance is not clear in practice [3]. Therefore, this review will focus on candidaemia uncomplicated by the presence of DSC.

A key determinant of successful mycological clearance is the pharmacokinetic/pharmacodynamic (PK/PD) profile of the antifungal agent, which links drug concentrations in plasma and tissues to microbiological efficacy and safety [28]. PK/PD parameters, such as volume of distribution, tissue penetration and duration of exposure, are essential for identifying safe antifungal dosing regimens that provide a high probability of microbiological efficacy in relation to the likely susceptibility of the infective organism [25]. Agents with a large volume of distribution and strong tissue affinity are generally more effective in treating deep‐seated and disseminated infections. Antifungals that maintain concentrations above the minimum inhibitory concentration (MIC) for prolonged periods of time or those that achieve high peak levels relative to MIC (concentration‐dependent activity) are more likely to clear infections effectively [25]. For echinocandins, both area under the curve/MIC (AUC/MIC, total exposure) and maximum serum concentration/MIC (*C*_max_/MIC, peak concentration) are linked to treatment success, with some data suggesting that optimising peak levels may offer an additional benefit [29].

Early mycological clearance is a well‐established concept in the management of other fungal infections, such as cryptococcal meningitis, where it is defined as the achievement of negative cryptococcal cerebrospinal fluid cultures within 2 weeks of initiating antifungal therapy [30, 31, 32]. However, candidaemia differs greatly from cryptococcal meningitis, particularly in critically ill or immunocompromised patients. Defining early mycological clearance in candidaemia might support healthcare professionals (HCPs) in refining management strategies and enhancing patient outcomes by providing a clear benchmark to aim for in clinical practice [33].

In the STRIVE and ReSTORE trials for rezafungin, early mycological clearance was defined as *Candida‐*negative blood cultures within 24 or 48 h, as well as by Day 5 of antifungal therapy [7], whereas a retrospective real‐world analysis suggests that achieving candidaemia clearance within 2 days of therapy initiation could be a realistic and suitable benchmark [34]. Identifying and utilising reliable prognostic markers of treatment response, such as time‐to‐positive (TTP) blood cultures, polymerase chain reaction (PCR) and (1,3)‐β‐d‐glucan (BDG) levels, may further aid in assessing mycological clearance in candidaemia. These markers may offer valuable insights into the effectiveness of antifungal treatment, optimise therapeutic decision‐making and help predict patient outcomes by supporting timely interventions.

### 2.1 TTP Blood Culture

A 2024, cross‐sectional, observational study investigating the role of TTP as a prognostic marker in a cohort of 7447 ICU patients observed that among 57 patients with candidaemia, the median TTP for deceased patients was 24 h compared with 35 h for survivors (*p* = 0.001) [16]. Logistic regression analysis revealed that each 1‐day reduction in TTP was associated with a 1.397‐fold increase in the odds of 30‐day mortality (95% CI, 1.08–1.741) [16], likely associated with a higher circulating fungal burden requiring prompt and efficacious treatment. This may potentially lead to a rapid reduction in burden and early clearance.

### 2.2 Diagnosis Through Polymerase Chain Reaction, T2 Magnetic Resonance and 1,3‐β‐d‐Glucan

Rapid and accurate diagnosis of candidaemia and IC remains a critical challenge in clinical practice, particularly in critically ill and immunocompromised patients. Traditional culture‐based methods often lack sensitivity and are limited by delayed turnaround times, prompting the need for more timely and reliable diagnostic tools [35]. Several non‐culture‐based approaches have emerged to address this gap, including PCR, T2 magnetic resonance (T2MR) and BDG testing [16, 36, 37]. Integrating these technologies into clinical workflows may enhance early diagnosis, guide antifungal stewardship and improve outcomes in patients at risk for IC.

PCR‐based assays offer rapid detection of *Candida* DNA directly from blood samples and demonstrate high sensitivity and specificity, thus improving early detection, with variable species differentiation [36, 38]. However, further studies are needed to validate the clinical usefulness of PCR diagnostics in candidaemia management [38].

T2MR integrates magnetic resonance and nanotechnology, allowing for the accurate detection of whole *Candida* species cells. This method achieves high sensitivity and specificity without requiring prior isolation of *Candida* cells, even when positive blood cultures are not present, and can identify *Candida* species in just 3–5 h [37]. Importantly, initial in vitro studies on exogenous substances have demonstrated that the T2MR assay remains unaffected by antifungal agents [37]. A multicentre trial (*N* = 31 patients with positive *Candida* blood samples) showed that the T2MR assay detected candidaemia in 18.2% of patients by the end of the first week, while blood cultures showed 0%. The T2MR test significantly improved post‐treatment surveillance over conventional cultures (Chi‐square = 8.2; *p* = 0.004). The study concluded that the T2MR assay is superior to conventional blood cultures for monitoring candidaemia clearance during antifungal treatment, with significant implications for assessing treatment duration and source control [37]. A different multicentre study (*N* = 49 adults receiving empirical antifungal therapy for suspected IC) found that T2MR may help identify patients at risk of poor outcomes. A positive T2MR was associated with a higher risk of a poor outcome (35.7% versus 0.0%; *p* = 0.0001). The specificity and positive predictive value (PPV) of a positive T2MR for predicting poor outcomes were 100%, with a negative predictive value of 79.6%. However, further studies are required to assess the viability of *Candida* species cells detected by the T2MR assay and to evaluate the impact of T2MR on diagnosis, treatment, patient outcomes and healthcare costs [37, 39].

BDG, a pan‐fungal cell wall component, serves as a broad biomarker for invasive fungal infections, offering a high negative predictive value but limited capacity to identify *Candida* alone [36, 40, 41]. A multicentre, randomised trial indicated that a declining BDG level is linked to successful treatment outcomes, with a PPV of 90%, while increasing BDG levels correspond to treatment failure, exhibiting a negative predictive value (NPV) of 90% [39]. However, it is crucial to recognise that 17.6% of patients with candidaemia may persistently test negative for BDG [40]. Those patients showed higher mycological clearance rates, fewer complications and a lower 30‐day mortality rate, suggesting that the predictive value of BDG may be limited [40]. The main limitation of BDG testing is that positivity is associated with a range of fungal infections and is not specific to IC [42]. False‐positive results can occur, due to the presence of BDG in the bloodstream due to non‐infective sources, including IV immunoglobulin and albumin therapy, gauze packing, IV amoxicillin/clavulanic acid, the use of cellulose depth filters and mucosal barrier injury resulting in the translocation of intestinal luminal BDG [42, 43].

Therefore, BDG, PCR and T2MR testing all show limitations, not only as diagnostic markers for invasive fungal infections such as candidaemia but also as markers of early fungal clearance.

### 2.3 Definition of Early Mycological Clearance in Uncomplicated Candidaemia

Despite their slower turnaround times, blood cultures remain the gold standard diagnostic test for candidaemia, highlighting the need for more efficient diagnostic tests for candidaemia [2]. Slower turnaround times limit clinical utility and particularly impact the ability for early treatment of suspected candidaemia. Given its complex nature, no clinical marker should be used in isolation to diagnose or assess the severity of candidaemia. Current evidence suggests that a TTP blood culture may serve as a valuable surrogate marker for microbial load and infection severity in candidaemia, but it should only be used alongside other clinical signs and symptoms to determine clinical management. Blood BDG negativity may indicate a lower risk of adverse mortality outcomes; however, data do not support its use as a sole predictor of microbial response and outcome due to its poor specificity. As such, BDG testing should be used with caution; therefore, currently BDG positivity should not be included in the definition of uncomplicated candidaemia or early mycological clearance. PCR testing offers high sensitivity and specificity, which may facilitate an earlier diagnosis of candidaemia compared with blood cultures. However, robust data are lacking on whether PCR‐based testing can positively impact patient outcomes.

**FIGURE 1:**
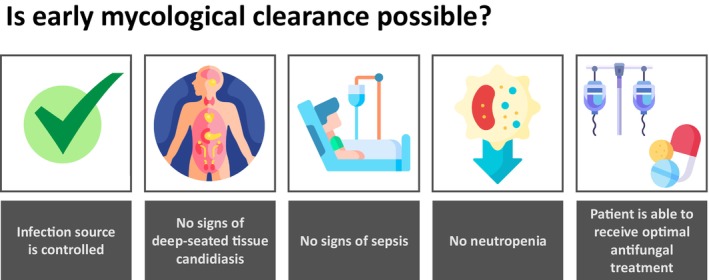
Criteria for defining early mycological clearance in uncomplicated candidaemia.

We therefore propose the following definitions for uncomplicated candidaemia and its early mycological clearance:

- Uncomplicated candidaemia can be defined as a *Candida*‐positive blood culture in patients with source control (e.g., removal of central venous catheters [CVCs], resection of abdominal abscesses and other successful surgical procedures), no signs of sepsis, no significant immunosupression, with early mycological clearance and no signs of DSC (Table 1)
- Early mycological clearance of uncomplicated candidaemia in all patients, including those who may be immunocompromised, can be defined as the blood culture clearance of *Candida* species achieved with optimal antifungal treatment and an appropriate loading dose (to be determined by the clinician) within 3 days of the first positive blood culture (Figure 1)

**TABLE 1:** Key principles for managing uncomplicated candidaemia.

Principles of uncomplicated candidaemia management	
Monitoring *Candida* clearance	Blood or tissue cultures are the gold standard for monitoring clearance of *Candida* spp. BDG and PCR results should be interpreted with caution and alongside blood or tissue culture results
Source control	Infection sources, such as CVCs, should be removed as soon as practically possible
Management	Echinocandins should be used as the first‐line treatment option for candidaemia Clinicians should use a ‘hit early and hard’ approach to treating candidaemia The treatment goal is a negative blood *Candida* culture within 3 days of the first blood‐positive culture Antifungal treatment should be continued for 14 days after a negative *Candida* blood culture Clinicians should avoid underdosing of antifungals, such as echinocandins, to reduce the emergence of resistant *Candida* strains

Abbreviations: BDG, (1,3)‐β‐d‐glucan; CVC, central venous catheter; PCR, polymerase chain reaction.

Multiple factors, including a patient's immune status, can impact a patient's ability to clear uncomplicated candidaemia; therefore, HCPs should use these definitions as benchmark goals and not as a definitive determinant of treatment success, infection severity or outcome.

## 3 The Impact of Early Mycological Clearance

Despite its potential clinical importance, the relationship between early mycological clearance and patient outcomes in candidaemia remains underexplored in the literature. This represents a critical gap in understanding, particularly in the context of growing antifungal resistance, rising healthcare costs and the urgency of optimising treatment strategies. Achieving early clearance of *Candida* from the bloodstream may be a key indicator of early, effective antifungal therapy and immune recovery, and is likely associated with reduced risk of secondary organ involvement, lower mortality rates and shorter durations of both intensive care and overall hospitalisation. Early control of infection may be of particular importance in infections with resistant species such as *Candida auris*.

In addition to improving clinical outcomes, early clearance may contribute to more efficient resource utilisation by enabling earlier de‐escalation or discontinuation of antifungal therapy and reducing the need for extensive diagnostic or interventional procedures. From a stewardship perspective, it offers an opportunity to limit antifungal exposure, thereby reducing the risk of toxicity, resistance development and costs associated with prolonged therapy. However, due to variability in definitions, timing of culture sampling and study endpoints, the true prognostic value of early clearance remains uncertain. Further prospective studies are warranted to clarify its role as a therapeutic target and potential surrogate marker in both research and clinical practice.

### 3.1 Patient Outcomes

Persistent candidaemia may be a risk factor for mortality in patients with candidaemia. In an observational, retrospective, multicentre study of patients with candidaemia (*N* = 1188) from three hospitals in Italy and Spain, persistent candidaemia, defined as ≥ 1 positive blood culture with the same *Candida* species ≥ 5 days from the start of active antifungal treatment, was associated with higher 30‐day mortality (aHR 1.605, 95% CI 1.176–2.191, *p* = 0.003) [44]. *Candida parapsilosis* (sHR 1.312, 95% CI 1.075–1.633, *p* = 0.03) and *Candida auris* (sHR 1.549, 95% CI 1.155–2.159, *p* = 0.029), compared with *Candida albicans* , were associated with an increased risk of persistent candidaemia [44]. Patients with persistent candidaemia required a longer duration of antifungal treatment than those without, which resulted in extended hospital stays and higher healthcare expenses [44]. These findings highlight the importance of early and effective antifungal therapy and source control for both patients and clinical settings, particularly in patients with difficult‐to‐treat and persistent *Candida* infections caused by *Candida parapsilosis* and *Candida auris*.

A retrospective study, conducted in the United States and involving 827 patients, indicated that early mycological clearance correlates with enhanced real‐world outcomes and improved healthcare resource utilisation [34]. Most patients had bloodstream infections caused by *Candida albicans* (34.8%) or *Candida glabrata* (20.2%) [34]. A relationship emerged between the duration of candidaemia, measured by the day of the last positive blood culture, and in‐hospital mortality: patients with candidaemia lasting 1 day or less exhibited significantly lower in‐hospital mortality rates compared with those with 2 or more days of positive blood cultures (*p* = 0.046) or those with 3 or more days of positive blood cultures (*p* = 0.043) [34]. These initial findings highlight the potential advantages of achieving early mycological clearance, particularly in improving outcomes for critically ill patients in the ICU setting.

Furthermore, a subgroup analysis from the ReSTORE trial (including the China extension phase) showed that in 84 and 88 patients with candidaemia in the rezafungin and caspofungin arms, respectively, a greater percentage of patients achieved mycological eradication by day 5 with rezafungin (77.4%) compared with caspofungin (68.2%) [45]. For the candidaemia‐only group, patients who achieved day 5 mycological eradication and received rezafungin had a numerically lower day 30 all‐cause mortality rate (21.5%) compared with those treated with caspofungin (28.3%) [45]. Among those discharged from the ICU during the trial, the median length of stay in the ICU was 9 days shorter in patients treated with rezafungin compared with those treated with caspofungin [45]. The authors attribute these differences to the differentiated PK/PD profile of rezafungin and its front‐loaded dosing [45]. This subgroup analysis illustrates the potential advantages of rezafungin in achieving early mycological clearance, which may, in turn, reduce the mortality associated with candidaemia.

### 3.2 Economic Burden and Hospital Stay

The treatment of candidaemia represents a significant financial burden for healthcare systems, posing substantial economic challenges [46]. Treatment costs are linked to drug acquisition costs as well as drug effectiveness [47]. A systematic analysis of global study data revealed that the mean total cost per patient with candidaemia and IC can range from $48,487 up to $157,574 [48]. The cost of candidaemia is primarily driven by the expenses of extended length of hospital stays in addition to antifungal medication costs [3, 22, 48].

A health economic study suggested that administering rezafungin, compared with other echinocandins, could result in an average saving of €7175 per hospital case of IC or candidaemia, primarily due to shorter ICU stays as a result of earlier mycological clearance as observed in the STRIVE study [46]. Hybrid modelling, including a short‐term decision tree and a long‐term Markov model, demonstrated that weekly rezafungin was cost‐saving compared with daily echinocandin, achieving discounts of £3863, £4209 and £4586 compared with caspofungin, micafungin and anidulafungin, respectively. Again, costs were driven by differences in hospital stay duration between the treatments [49].

Analysis of pooled data from STRIVE and ReSTORE demonstrated that weekly rezafungin resulted in shorter mean hospital (25.2 days vs. 28.3 days, respectively) and ICU stays (16.1 vs. 21.6 days, respectively) compared with daily caspofungin when treating IC or candidaemia [50]. If a once‐weekly echinocandin option had been available, principal investigators would have considered an earlier discharge than the actual discharge date for 16% of patients in the ReSTORE trial [50]. However, it is unclear whether the reductions in ICU duration and hospital stay were directly attributable to the early mycological clearance associated with the use of rezafungin.

In a multi‐country retrospective case series of 15 patients (14 with IC and one with chronic pulmonary aspergillosis), five patients (36%) achieved a complete response, while seven (50%) showed a partial clinical, radiological or mycological response at day 30 post‐IC diagnosis [51]. Rezafungin administration reduced the need for central venous line placement, minimising potential associated complications, such as infections or thrombosis, and lowered daily costs related to human resources [51]. Therefore, rezafungin was selected by 86% of healthcare providers to enable outpatient parenteral antifungal therapy [51]. While source control remains essential in managing DSC, rezafungin may also improve health‐related quality of life and assist in symptom control [51]. This real‐world study highlights the potential benefits of rezafungin for patients with invasive candidiasis.

A retrospective, US‐based, observational study investigated the relationship between early candidaemia clearance and hospital stay by reviewing records of 867 patients with candidaemia [33, 34]. The study found that for each additional day of candidaemia, the odds of in‐hospital mortality increased by 3% (*p* = 0.019), with patients with ≥ 6 days of candidaemia having an increased risk of mortality of 14.0%, The median hospital length of stay post‐index collection day increased by 1.0 days (*p* < 0.001) and median total costs post‐index collection day increased by $3006 (*p* < 0.001) [34].

These data highlight the potential benefits of early mycological clearance of uncomplicated candidaemia for both patient outcomes and healthcare treatment costs, and warrant further investigation to better understand the broader implications of early mycological clearance in the management of candidaemia. Early treatment of candidaemia following a ‘hit early and hard’ approach may facilitate early mycological clearance of systemic candidiasis and reduce hospital stays, morbidity and mortality. However, the authors believe that further data, including country‐specific cost analyses, are needed to assess the optimal timeframe for clearance to achieve clinically meaningful patient outcome improvements and substantial treatment cost reductions.

## 4 Resistance to Antifungal Therapy and Early Mycological Clearance

Antifungal‐resistant *Candida* species represent a growing threat to patient outcomes, complicating the treatment of IC and leading to higher morbidity, prolonged hospital stays and increased healthcare costs [52, 53]. *C. parapsilosis* has naturally occurring polymorphisms in the BDG *FKS* subunit, altering its susceptibility to echinocandins and necessitating higher doses to achieve fungicidal activity [54]. Generally, in adults, echinocandins are given at a fixed dose regardless of body weight or *Candida* species susceptibility; therefore, underdosing of echinocandins in obese patients, along with poor penetration, may be partially responsible for the emergence of echinocandin resistance [17, 55, 56]. Insufficient dosing of triazoles has been associated with the emergence of antifungal resistance, including fluconazole and voriconazole resistance in *C. parapsilosis* species and increasing rates of echinocandin resistance in *C. glabrata* species [18, 57, 58]. This highlights the critical importance of optimising antifungal dosing and achieving early control of infection, particularly in resistant species such as *Candida auris*. Therefore, high‐dose, cidal antifungal regimens, such as rezafungin, may help mitigate the proliferation of resistant strains, improve patient outcomes and potentially reduce the costs associated with treating resistant infections [17, 54].

Liposomal amphotericin B is a broad‐spectrum polyene effective against fungi such as *Candida*, and an alternative for the treatment of invasive candidiasis, including in ICU settings [59, 60]. This is because it has low MIC resistance patterns across most *Candida* spp., minimal drug interactions, a good ability to overcome biological barriers, no requirement for drug monitoring and a favourable safety profile. However, amphotericin B can be associated with more side effects than echinocandins [60, 61].

Emerging data indicate that echinocandins, such as rezafungin, may promote early mycological clearance of *Candida* [7, 24, 45], which could lower the likelihood of resistant fungal strains emerging, in contrast to other types of antifungals. Animal model studies on PK/PD optimisation for treating resistant *FKS*‐mutated *Candida* isolates found the same mutant prevention concentration (MPC) for both rezafungin and micafungin [17, 62]. However, a wider safety margin and increased plasma exposures, which allow dosing towards the MPC to prevent resistance development, were achieved by rezafungin. These existing preliminary data highlight the potential for rezafungin in either treating or preventing echinocandin resistance in clinical practice [17, 62]. Early clearance of candidaemia with a ‘hit early and hard’ approach may be instrumental in preventing the emergence of resistance, but further data are required.

## 5 Challenges and Future Directions

Despite the potential benefits of early mycological clearance, several challenges remain in the management of candidaemia. Delayed diagnosis, inadequate or inappropriate empirical antifungal therapy and inappropriate antifungal dosing can hinder the early clearance of uncomplicated candidaemia. Therefore, we strongly advise that clinicians prioritise early diagnosis, timely initiation of appropriate antifungal therapy and, where possible, removal of the infection source, such as CVCs, as soon as practically possible.

The emergence of multidrug‐resistant *Candida* species, such as *C. parapsilosis* and *Candida auris*, poses a significant challenge to early clearance efforts [18, 63]. Clinicians must recognize that insufficient dosing of antifungals not only increases healthcare costs and jeopardizes patient outcomes but may also accelerate the development of antifungal resistance, posing a significant threat to effective treatment strategies. It is imperative that antifungal dosing schedules are followed and completed in full to reduce the risk of the development of resistant *Candida* strains. Antifungal treatment should be continued for 14 days after a negative *Candida* blood culture to ensure the infection is cleared and to avoid deep‐organ involvement [18].

Future research should focus on improving diagnostic technologies to detect candidaemia efficiently and rapidly, and on improving our ability to determine mycological clearance and identify refractory disease. Early mycological clearance in candidaemia as a primary goal should be incorporated into clinical practice recommendations when sufficient medical evidence demonstrates a clear benefit in patient outcomes and reduction of the economic burden of the disease [16, 19].

Existing data highlight the potential benefits of rezafungin in achieving early mycological clearance, optimising patient outcomes and reducing the risk of the emergence of echinocandin‐resistant *Candida* strains [7, 17, 18, 22, 24, 45, 62]. Additionally, animal studies suggest that rezafungin may aid in treating or preventing echinocandin resistance [17, 62]. However, further research is required to fully understand its role in these processes.

## 6 Conclusions

**FIGURE 2:**
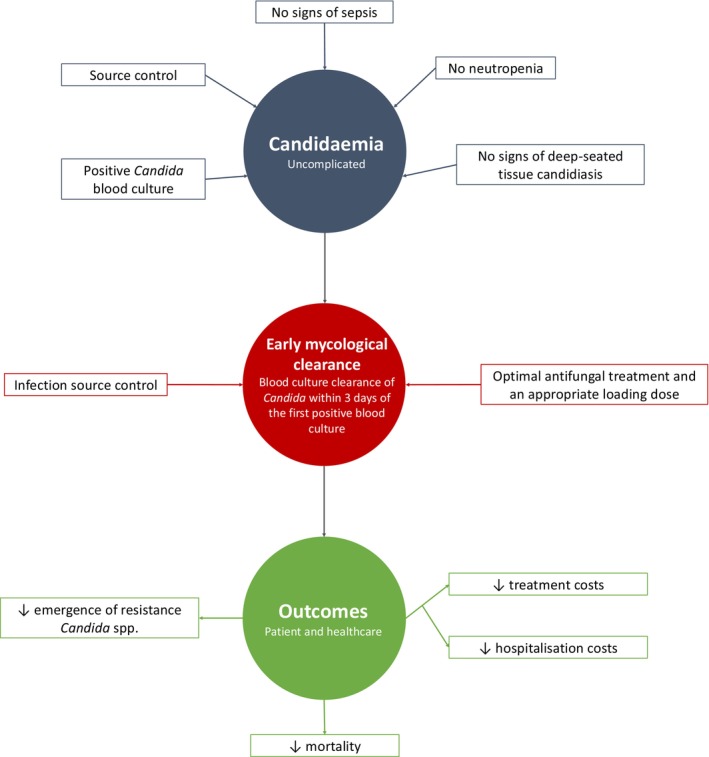
Principles for defining early mycological clearance in uncomplicated candidaemia and the potential benefits for clinical practice.

Emerging evidence suggests that in candidaemia, rapid eradication of fungal pathogens may shorten the required duration of antifungal therapy and improve clinical outcomes (Figure 2). However, further research is needed to improve the diagnosis of candidaemia and understand the relationship between the timing of mycological clearance and its impact on patient prognosis. In particular, studies should focus on identifying the clearance time point that correlates most strongly with improved outcomes, which may help establish an optimal threshold for treatment duration.

Echinocandins remain the first‐line therapy for most cases of candidaemia due to their favourable safety profile and fungicidal activity against *Candida* species. However, there is growing concern that underdosing in critically ill patients—due to altered pharmacokinetics, volume of distribution or obesity—may limit their effectiveness in achieving early fungal clearance. This underscores the need for trials that evaluate optimal dosing strategies in real‐world ICU populations.

Challenges to achieving early clearance continue, including delays in diagnosis and treatment, difficulties in timely CVC removal and the lack of reliable early prognostic markers. Improving outcomes will depend on prioritising early and accurate diagnosis, rapid initiation of effective antifungal therapy and leveraging advanced diagnostic technologies. These steps can support faster pathogen clearance, improve patient outcomes and ultimately reduce healthcare costs associated with prolonged or inadequate treatment.

## Author Contributions

**O. A. Cornely:** writing – review and editing, writing – original draft. **M. Bassetti:** writing – review and editing. **C. Garcia‐Vidal:** writing – review and editing. **M. Hoenigl:** writing – review and editing. **J. Maertens:** writing – review and editing. **I. Martin‐Loeches:** writing – review and editing. **J. P. Mira:** writing – review and editing. **P. L. White:** writing – review and editing.

## Funding

This work was supported by Mundipharma Research Limited.

## Conflicts of Interest

C.G.‐V. has delivered sponsored talks for AbbVie, AdvanzPharma, Avir Pharma, Basilea, Gilead, Janssen, MSD, Mundipharma, Pfizer, Sanofi and Shionogi; has served on advisory boards for AdvanzPharma, Gilead, MSD, Mundipharma, Pfizer and Shionogi; and has received grants from Gilead and Mundipharma. I.M.‐L. reports receiving lecture fees from Gilead, Thermofisher, Pfizer, Menarini and MSD, and has served on advisory boards for Gilead and Mundipharma. J.M. reports receiving grants or contracts from F2G, Gilead, MSD, Mundipharma, Pfizer; consulting fees from Abbvie, Asahi Kasei Pharma Corporation, Basilea, Cidara, Daiichi Sankyo, Elio Therapeutics, Gilead, MedPace, Menarini, Mundipharma, Novo Nordisk, Pfizer, Scynexis, Servier, Shionogi and Takeda; and speaker and lecture honoraria from Takeda, Abbvie, Gilead, Medscape/WebMD, MSD. J.P.M. has received fees for lectures from Astellas, MSD and Mundipharma; is a member of the expert boards for Astellas, Gilead, MSD and Mundipharma; and has received funding to attend national and international congresses from Astellas, MSD, Mundipharma and Pfizer. P.L.W. has performed diagnostic evaluations and received meeting sponsorship from Associates of Cape Cod, Bruker, Dynamiker and Launch Diagnostics; has performed diagnostic evaluations for IMMY and Virclia; has received speaker fees, expert advice fees and meeting sponsorship from Gilead and Mundipharma; has received speaker and expert advice fees from Pfizer and expert advice fees from F2G. M.B. reports receiving advisory board, speaker and research fund fees from Advanz, Cidara, Gilead, Menarini, MSD, Mundipharma, Pfizer and Shionogi. M.H. has received research funding from Astellas, Basilea, F2G, Gilead, GSK, IMMY, Karius, Melinta, MSD, Mundipharma, Pfizer, Pulmocide, Scynexis and Shionogi. O.A.C. reports grants or contracts from BMBF, Cidara, DFG, DZIF, EU‐DG RTD, F2G, Gilead iHi, iMi, MedPace, MSD, Mundipharma, Octapharma, Pfizer, Scynexis; consulting fees from Abbvie, AiCuris, Basilea, Biocon, Boston Strategic Partners, Cidara, Elion Therapeutics, Gilead, GSK, IQVIA, Janssen, Matinas, MedPace, Menarini, Melinta, Molecular Partners, MSG‐ERC, Mundipharma, Noxxon, Octapharma, Pardes, Pfizer, PSI, Scynexis, Seres, Seqirus, Shionogi, The Prime Meridian Group; speaker and lecture honoraria from Abbott, Abbvie, Akademie für Infektionsmedizin, Al‐Jazeera Pharmaceuticals/Hikma, amedes, AstraZeneca, Deutscher Ärzteverlag, Gilead, GSK, Grupo Biotoscana/United Medical/Knight, InfectoPharm, Ipsen Pharma, Medscape/WebMD, MedUpdate, MSD, Moderna, Mundipharma, Noscendo, Paul‐Martini‐Stiftung, Pfizer, Sandoz, Seqirus, Shionogi, streamedup!, Touch Independent, Vitis; payment for expert testimony Cidara and participation on a DRC, DSMB, DMC, Advisory Board for AstraZeneca, Cidara, IQVIA, Janssen, MedPace, Melinta, PSI, Pulmocide, Vedanta Biosciences. O.A.C. is an editor of *Mycoses* but had no role in the review or decision process for this manuscript.

## Data Availability

Data sharing not applicable to this article as no datasets were generated or analyzed during the current study.
